# Supplementary material for: Silicon/Graphite/Amorphous Carbon as Anode Materials for Lithium Secondary Batteries
Source: Molecules. 2023 Jan 4;28(2):464. doi: 10.3390/molecules28020464 (PMC9865035; doi:10.3390/molecules28020464)
Supplement: Supplementary file 1 [file molecules-28-00464-s001.zip › molecules-2093786-supplementary.pdf]

Supporting information

Silicon/Graphite/Amorphous Carbon as Anode Materials for Lithium Secondary Batteries

Haojie Duan [1, 2], Hongqiang Xu [2], Qian Wu [2], Lin Zhu [2], Yuting Zhang [2], Bo Yin [2], Haiyong He [1, 2, *]

1 School of Materials Science and Chemical Engineering, Ningbo University, Ningbo 315211, China
2 Ningbo Institute of Materials Technology and Engineering, Chinese Academy of Sciences, Ningbo 315201, China
* Correspondence: hehaiyong@nimte.ac.cn

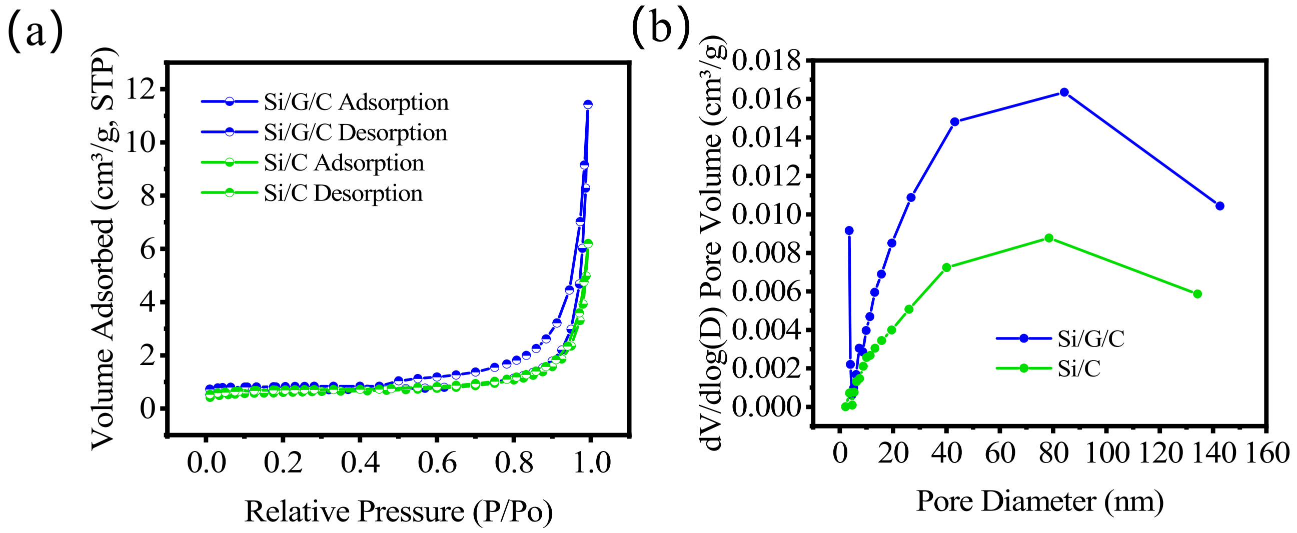

Figure S1. (a) N_2 adsorption–desorption isotherms, and (b) pore size distribution of Si/G/C and Si/C.

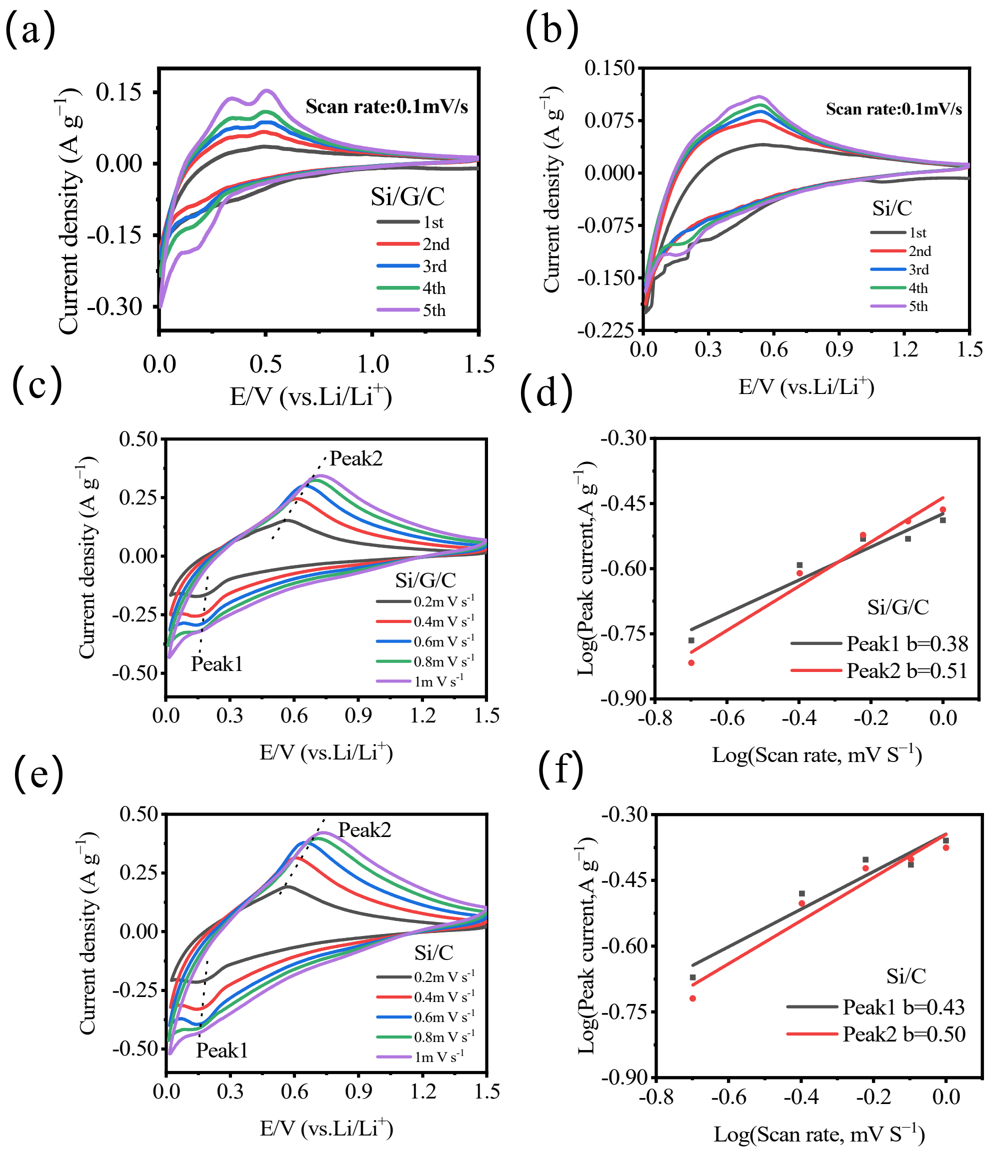

Figure S2. The initial five CV curves of the (**a**) Si/G/C and (**b**) Si/C at 0.1 mV s^{-1}, (**c**, **e**) CV curves at different scan rates, (**d**, **f**) linear fitting relationship between log i and log v at different redox peaks.

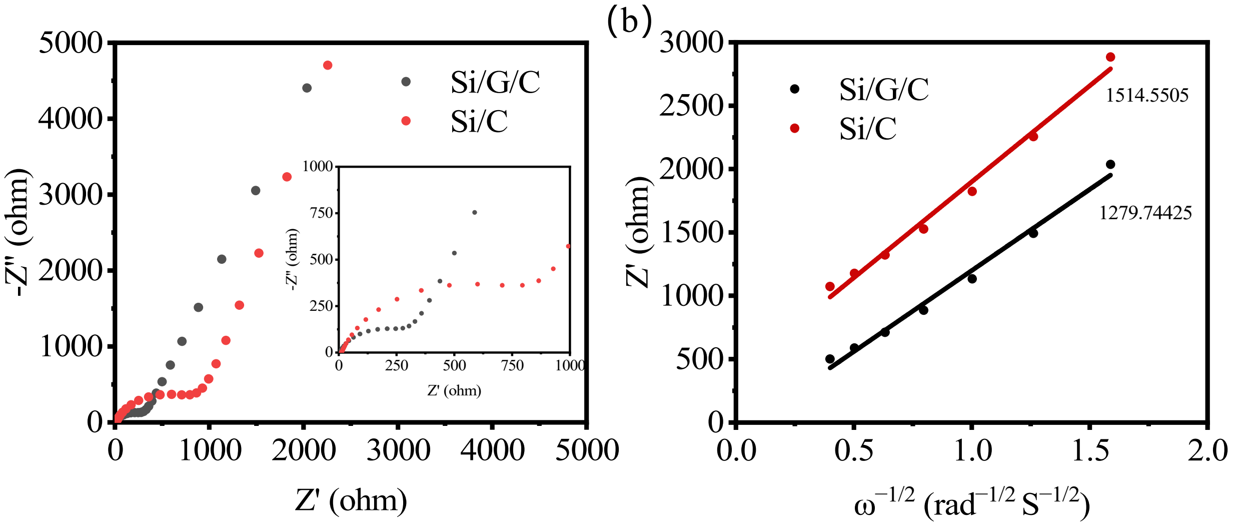

Figure S3. (**a**) electrochemical impedance spectra and (**b**) fitting Z′ and $\omega^{-1/2}$ of Si/G/C and Si/C.

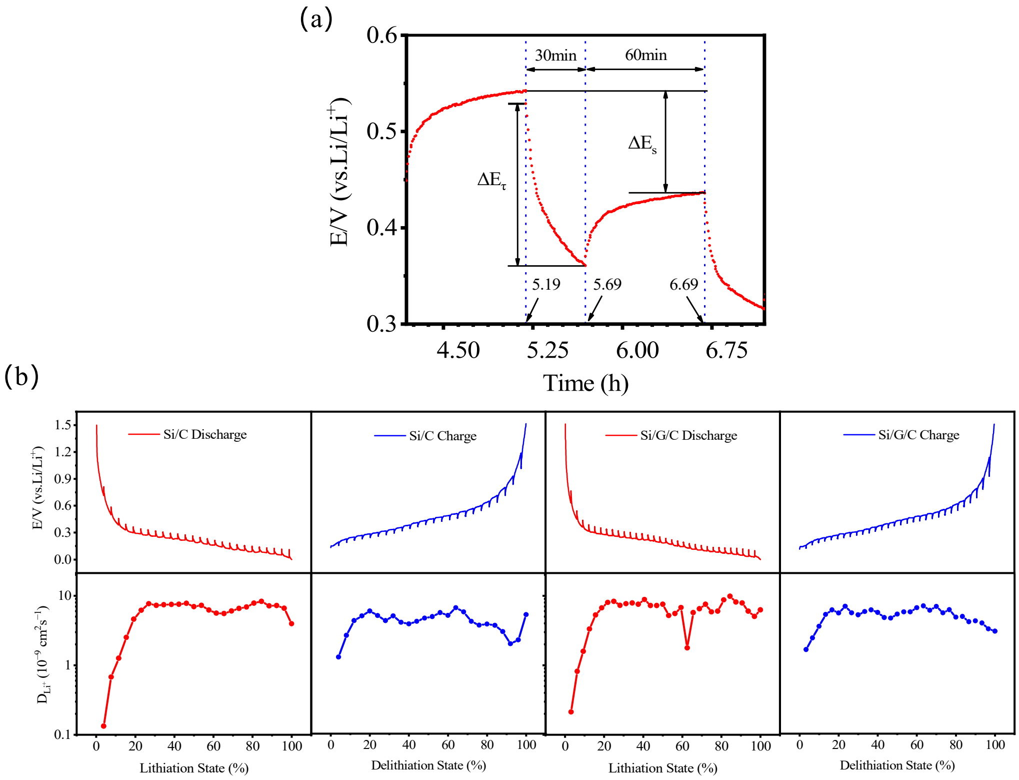

Figure S4. (**a**) E vs. t curves of Si/G/C electrode for a single discharge impulse and marked ΔE_S and ΔE_τ. (b) GITT curves and corresponding Li^+ diffusion coefficient at different lithiation/delithiation states of the Si/G/C and Si/C electrodes.

The lithium-ion diffusion coefficient was measured by GITT with a pulse current of 0.1 A g^{-1} for a pulse time of 30 min between 1 h rest intervals (Figure S4a) and calculated by Equation: $D = \frac{4L^2}{\pi\tau}(\frac{\Delta E_S}{\Delta E_\tau})^2$, where t is the duration of the imposed current pulse (s), τ represents the relaxation time (s), and ΔEs means the steady-state potential change (V) caused by the imposed current pulse. ΔEτ is the potential change (V) during the constant current pulse after eliminating the iR drop. L is lithium-ion diffusion distance (cm); here is approximately equal to the thickness of the electrode material.
